# Supplementary material for: A spatial regime shift from predator to prey dominance in a large coastal ecosystem
Source: Commun Biol. 2020 Aug 27;3:459. doi: 10.1038/s42003-020-01180-0 (PMC7452892; doi:10.1038/s42003-020-01180-0)
Supplement: Supplementary file 1 — Supplementary Information [file 42003_2020_1180_MOESM1_ESM.pdf]

SUPPLEMENTARY FIGURES AND TABLES

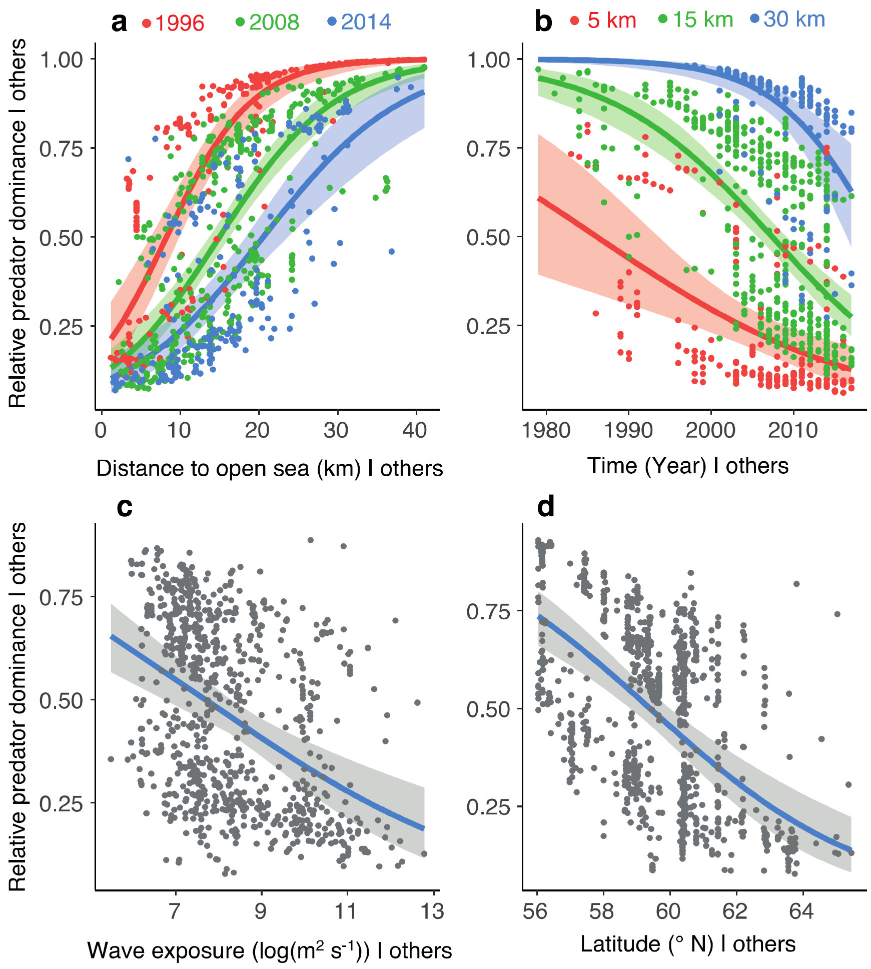

Supplementary Figure 1. Drivers of relative predator dominance. Partial residual plots showing the relative influence of **a**) distance to the open sea during three years (1996, 2008 and 2014), **b**) time (year) in three archipelago zones (outer, middle and inner archipelago), **c**) wave exposure, and **d**) latitude, on relative predator dominance. Slopes are best-fitting relations (± 95% confidence bands) and points are partial residuals.

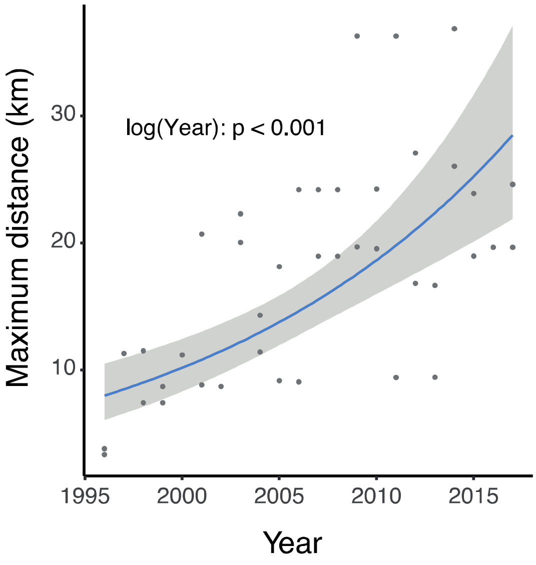

Supplementary Figure 2. Temporal change in the spatial extent of stickleback domination. Influence of sampling year (1995-2017) on the maximum distance from open sea to a stickleback-dominated bay (defined as relative predator dominance ≤0.10, that is ≥90% of fish are stickleback). Data from 1979-1994 was excluded because no sampling was conducted >20 km from the open sea (N = 46).

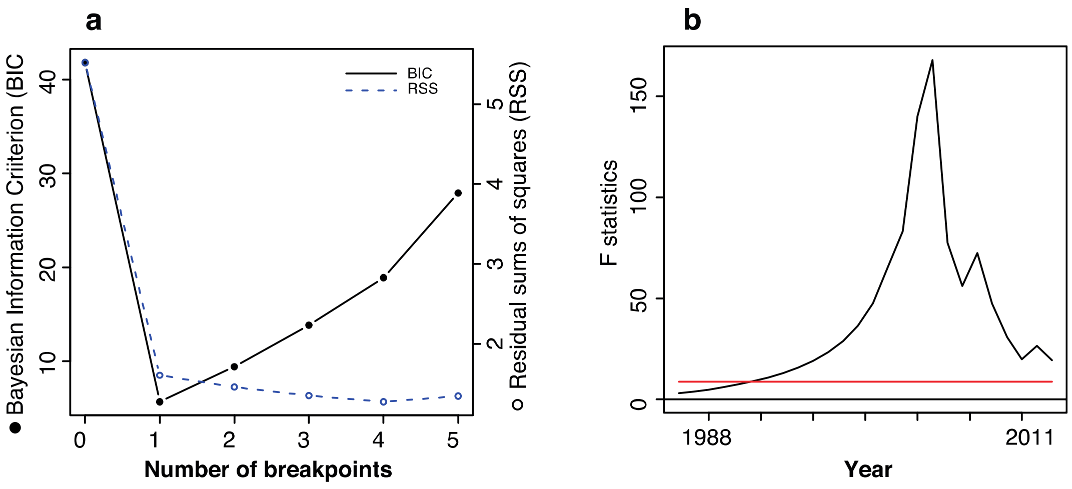

Supplementary Figure 3. Statistics from change-point analyses of the Forsmark time-series. Graphical representation of **a)** Bayesian Information Criterion (BIC; black circles) and residual sums of squares for models with 0-6 breakpoints (RSS; open circles and dashed line). ΔBIC of model with 1 breakpoint: 3.74. **b**) F statistic of individual years 1981-2017. The peak highlights 1 breakpoint in year 2004.

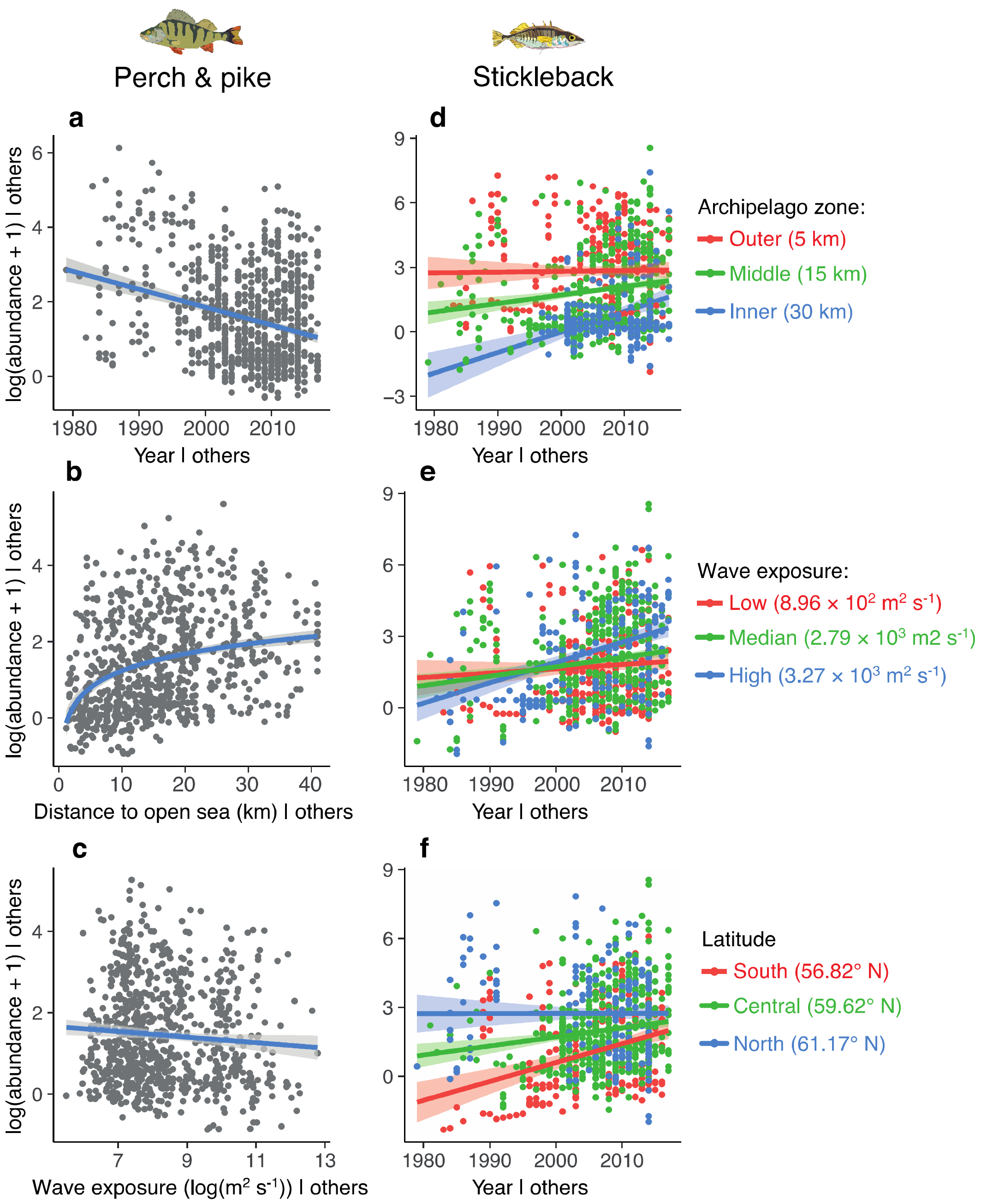

Supplementary Figure 4. Drivers of perch, pike and stickleback abundance. Partial residual plots displaying the relative influence of **a**) time, **b**) distance to open sea and **c**) wave exposure, on pooled perch and pike abundance. Right side: the relative influence of **d**) time × distance to open sea, e) time × wave exposure and e) time × latitude, on three-spined stickleback abundance. Note the different scales on the y-axes.

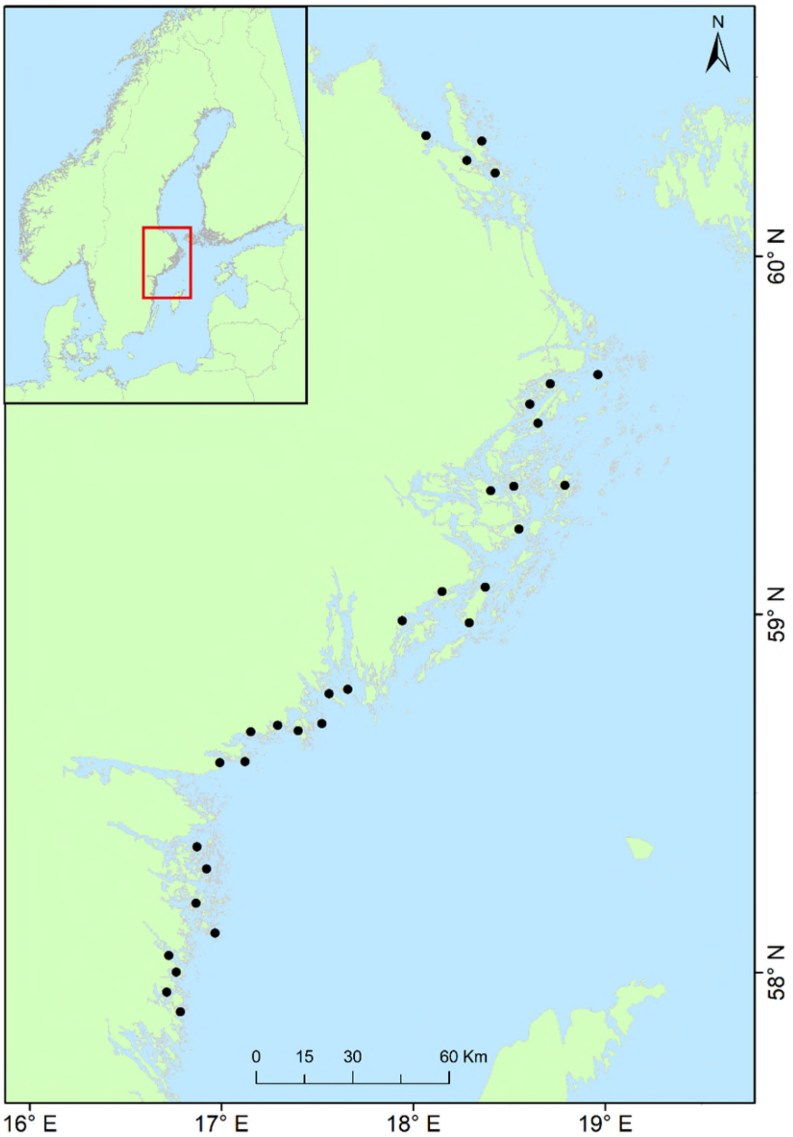

Supplementary Figure 5. Study area for 2014 ecosystem survey. Map over the central Swedish Baltic Sea coast, marking the position of the 32 bays sampled in spring (May) and summer (August) 2014.

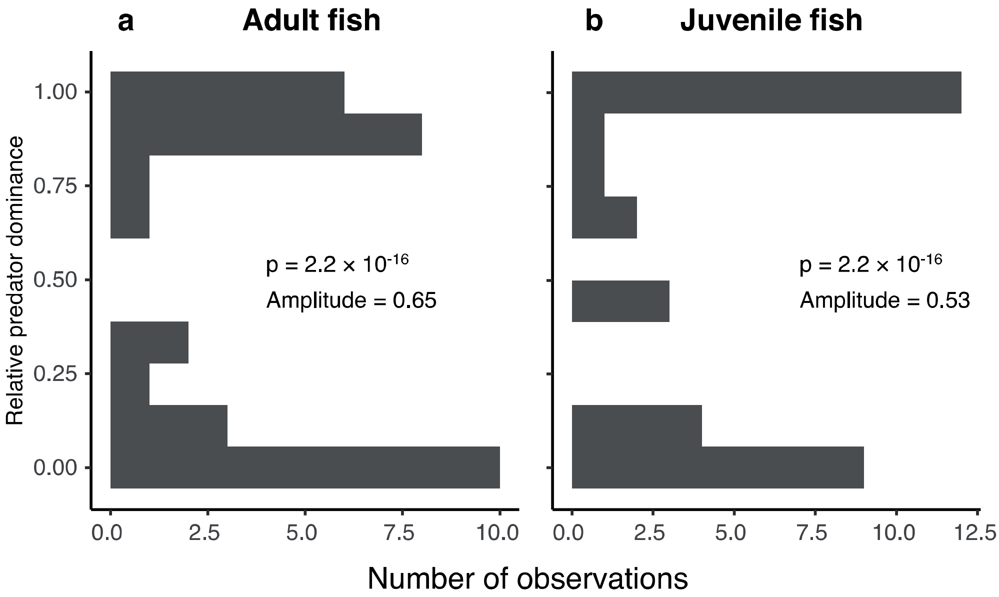

Supplementary Figure 6. Bimodal relative predator dominance in 2014 ecosystem survey. Frequency distribution of relative predator dominance (abundance of perch + pike / abundance of perch + pike + three-spined stickleback) in the 2014 ecosystem field survey for **a**) adults sampled in May and **b)** juveniles sampled in August (N = 31 bays). P-values are the likelihood of unimodality based on Hartigan's dip test, and amplitude (range: 0-1) the distinctness of modes. Moreover, adult and juvenile relative predator dominance are positively correlated (Spearman rank correlation; $r = 0.67$, $p<0.001$).

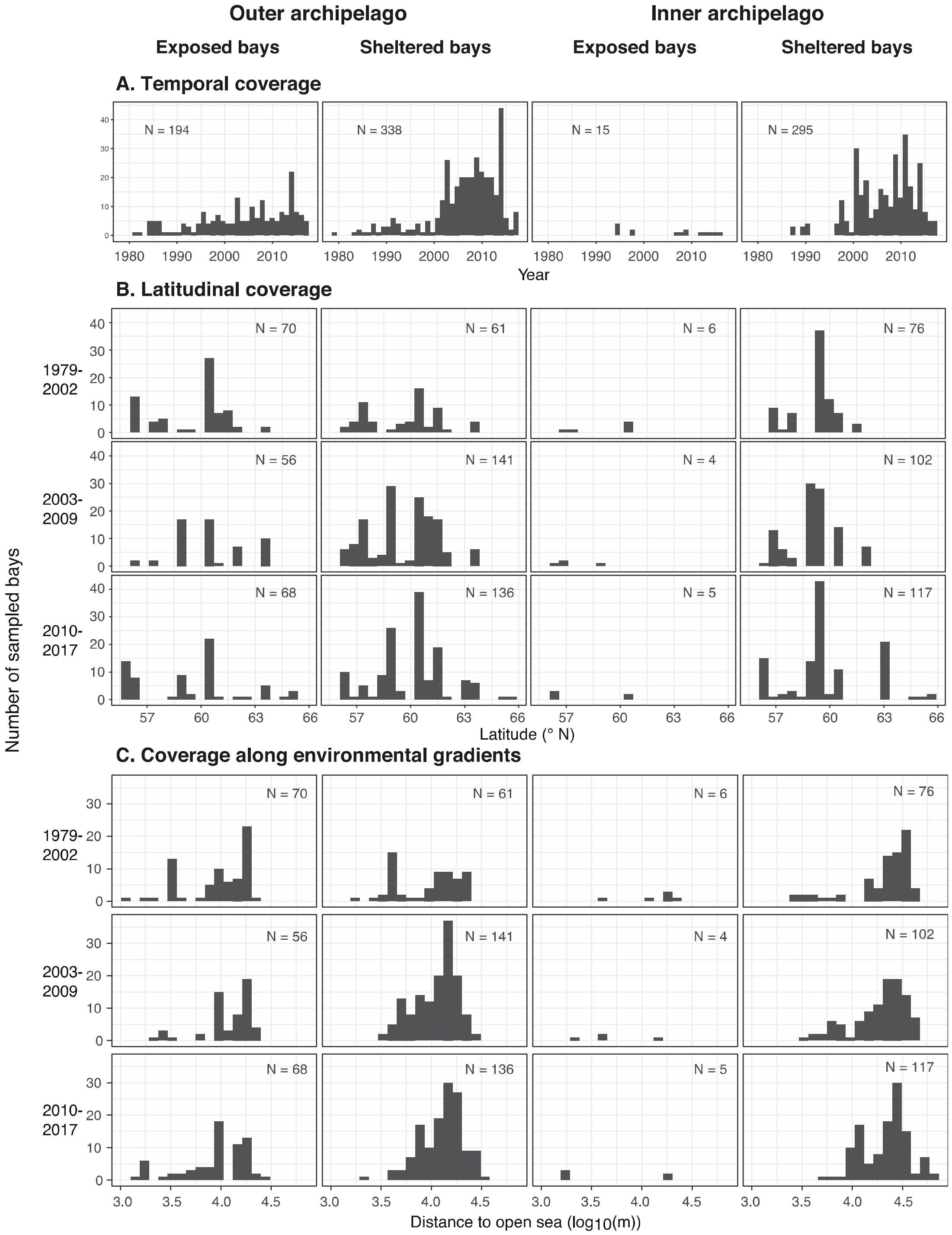

Supplementary Figure 7. Temporal and spatial coverage of juvenile fish sampling. Number of sampled bays **A**) per year (1979-2017) split by four archipelago types (outer vs. inner archipelago crossed with wave-exposed vs. wave-sheltered bays), **B)** across latitude, split by the same four archipelago types and three time periods (1979-2002, 2003-2009, 2010-2017), **C**) across distance to open sea, split by the four archipelago types and three time periods. Note that the figure includes the 9 predator-dominated bays occurring >41 km from the open sea, which were excluded prior to analyses.

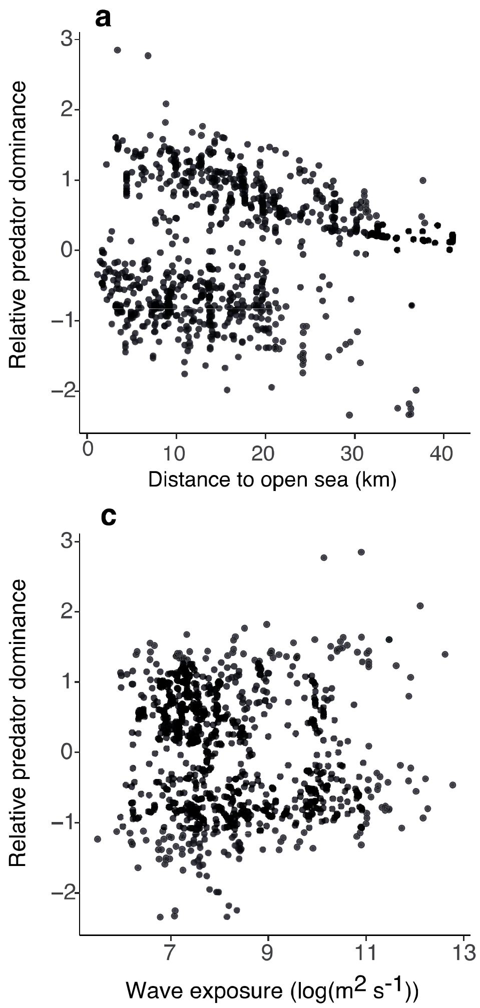

Supplementary Figure 8. Deviance residuals in relative predator dominance. Deviance residuals from the glm on factors explaining relative predator dominance in relation to **a**) distance to open sea (km), **b**) year, **c**) wave exposure and **d**) latitude (N = 833).

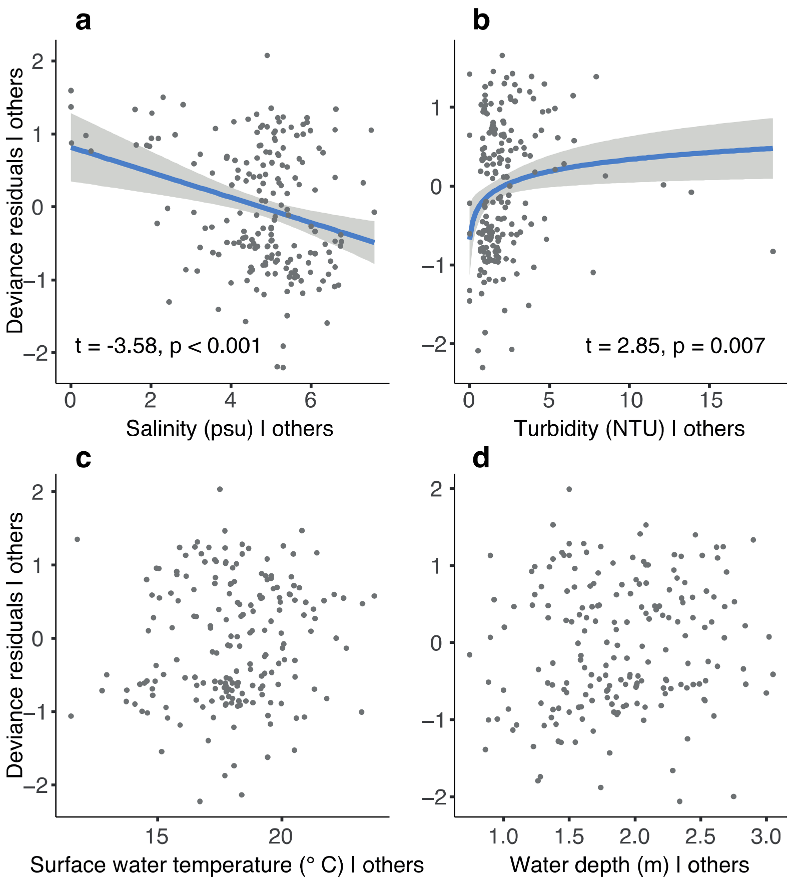

Supplementary Figure 9. Influence of local abiotic conditions on residual variability in relative predator dominance. Influence of **a**) salinity, **b**) turbidity, **c**) surface water temperature and **d**) depth on variability in the deviance residuals from the binomial generalized linear model on relative predator dominance (N = 192, $R^2 = 0.11$).

Supplementary tables

Supplementary Table 1. Summary of best-fitting general(ized) linear models. Summary of best-fitting glm's on spatial and temporal predictors of **a**) relative predator dominance, **b**) predator (perch+pike) abundance (log(x+1)), and **c**) stickleback abundance (log(x+1)).

**Parameter**	**Estimate**	**SE**	**z**	**p**	**R^2**
**a) Relative predator dominance**					0.24*
Intercept	104.7	45.30	2.312	**0.0208**	
Year (Y)	-4.296×10^{-2}	2.255×10^{-2}	-1.906	0.0567	
Distance (m) to open sea (D)	8.236×10^{-3}	3.263×10^{-3}	2.524	**0.0116**	
log(wave exposure)	-0.2895	0.060	-4.794	**<0.001**	
Latitude (L)	-0.305	0.047	-6.371	**<0.001**	
Y × D	-4.034×10^{-6}	1.625×10^{-6}	-2.482	**0.0130**	
**b) Predator abundance**	**Estimate**	**SE**	**t**	**p**	**0.17**
Intercept	92.152	11.523	7.994	**<0.001**	
Year	-0.048	0.006	-8.346	**<0.001**	
log(distance to open sea)	0.643	0.066	9.744	**<0.001**	
log(wave exposure)	-0.068	0.032	-2.14	**0.0325**	
**c) Stickleback abundance**	**Estimate**	**SE**	**t**	**p**	**0.25**
Intercept	-1519	508.8	-2.986	**0.003**	
Year (Y)	0.749	0.253	2.955	**0.003**	
Distance to open sea (D)	-0.007	0.002	-3.499	**<0.001**	
log(wave exposure) (W)	-37.32	11.58	-3.224	**0.001**	
Latitude (L)	31.00	8.696	3.565	**<0.001**	
Y × D	3.742×10^{-6}	1.083×10^{-6}	3.457	**<0.001**	
Y × W	0.0187	0.005	3.238	**0.001**	
Y × L	-0.015	0.004	-3.533	**<0.001**	

*: McFadden's pseudo R^2 for glm's.

Supplementary Table 2. Summary of candidate piecewise path analysis models. Model fit (p) to the data was assessed using the D-sep test. Note that p (model fit) < 0.05 means the model does not fit the data. Identification of the best-fitting model (model 13, marked in bold) was based on Akaike's Information Criterion for small samples (AICc). df = degrees of freedom, ΔAICc = difference in AICc units to the best-fitting model (#12). Ad. = Adult, Juv = juvenile, P = perch and pike, S = threes-spined stickleback.

#	Predictors	P	AIC	AICc	df	Δ AICc	Missing paths
1	Stock recruitment, distance to open sea, vegetation	0.064	62.36	138.083	20	77.161	AdP -> AdS, AdS-> JuvP
2	Stock recruitment, wave exposure, vegetation	**0.006**			16		AdP -> AdS, AdS-> JuvP; wave exp -> JuvS
3	Model 1 + missing paths	0.762	47.73	123.297	16	62.375	
4	Model 3 - effect of distance to open sea on AdS	0.752	47.64	113.605	18	52.683	
5	Model 4 - effect of spring vegetation on AdP	0.843	45.76	101.315	18	40.393	
6	Model 5 - P stock recruitment	0.874	44.72	92.417	22	31.495	
7	Model 6 - effect of summer vegetation on JuvS	0.854	44.87	86.932	24	26.01	
8	Model 2 + missing paths	0.895	45.89	129.324	14	68.402	
9	Model 8 - effects of wave exposure on AdP & AdS	0.867	45.6	108.741	18	47.819	
10	Model 10 - effect of spring vegetation on AdP	0.929	35.15	68.099	14	7.177	
11	Model 10 - JuvP stock recruitment	0.958	33.66	61.373	16	0.451	
**12**	**Model 11 - effect of summer vegetation on JuvS**	**0.878**	**35.37**	**60.922**	**18**	0	
13	Model 12 + effect of spring zooplankton on JuvP	0.703	44.04	80.312	22	19.39	
14	Model 13 - effect of adult stickleback on JuvP	0.161	54.74	94.28	24	33.358	AdS -> JuvP

Supplementary Table 3. Summary of best-fitting piecewise path analysis. Standardized path coefficients and p-values from the best-fitting model (model #12 in Table 1).

Response	Predictor	Estimate	P
$\log_{10}$(adult stickleback in spring)	$\log_{10}$(adult piscivores in spring)	-0.53	0.0014
$\log_{10}$(adult stickleback in spring)	√(total vegetation cover in spring)	0.31	0.043
$\log_{10}$(juvenile piscivores in summer)	√(rooted vegetation cover in summer)	0.49	0.0021
$\log_{10}$(juvenile piscivores in summer)	$\log_{10}$(adult stickleback in spring)	-0.45	0.0037
$\log_{10}$(juvenile stickleback in summer)	$\log_{10}$(adult stickleback in spring)	0.61	0.001
$\log_{10}$(juvenile stickleback in summer)	$\log_{10}$(wave exposure)	0.29	0.035

Supplementary Table 4. Bootstrapped drivers of relative predator dominance. Results from a bootstrapping procedure (500 iterations without replacement) to estimate the robustness of the best-fitting model on relative predator dominance, after excluding the repeated sampling (>1 years of data) in 131 (25%) of the 477 bays.

Parameter	Estimate	SD	SE
Intercept	84.1	20.5	0.916
Year (Y)	-0.0346	0.0103	0.462×10^{-3}
Distance to open sea (D)	7.81×10^{-3}	1.24×10^{-3}	55.4×10^{-6}
log(wave exposure)	-0.292	0.023	1.02×10^{-3}
Latitude	-0.236	0.013	5.69×10^{-4}
Y × D	3.83×10^{-6}	0.617×10^{-6}	0.027×10^{-6}
