# Supplementary material for: A spatial regime shift from predator to prey dominance in a large coastal ecosystem
Source: Commun Biol. 2020 Aug 27;3:459. doi: 10.1038/s42003-020-01180-0 (PMC7452892; doi:10.1038/s42003-020-01180-0)
Supplement: Supplementary file 4 — Description of Additional Supplementary Files [file 42003_2020_1180_MOESM4_ESM.pdf]

Description of Additional Supplementary Files

File Name: **Supplementary Data 1**
Description: Raw data used in the analyses underlying main figures 1-3 and 5.

File Name: **Supplementary Data 2**
Description: Raw data used in the analyses underlying main figure 4.
