# Supplementary material for: A spatial regime shift from predator to prey dominance in a large coastal ecosystem
Source: Commun Biol. 2020 Aug 27;3:459. doi: 10.1038/s42003-020-01180-0 (PMC7452892; doi:10.1038/s42003-020-01180-0)
Supplement: Supplementary file 5 — Reporting Summary [file 42003_2020_1180_MOESM5_ESM.pdf]

nature research

Corresponding author(s): Johan Eklöf

Last updated by author(s): Jul 3, 2020

Reporting Summary

Nature Research wishes to improve the reproducibility of the work that we publish. This form provides structure for consistency and transparency in reporting. For further information on Nature Research policies, see Authors & Referees and the Editorial Policy Checklist.

Statistics

For all statistical analyses, confirm that the following items are present in the figure legend, table legend, main text, or Methods section.

n/a	Confirmed	
☐	☒	The exact sample size (n) for each experimental group/condition, given as a discrete number and unit of measurement
☐	☒	A statement on whether measurements were taken from distinct samples or whether the same sample was measured repeatedly
☐	☒	The statistical test(s) used AND whether they are one- or two-sided<br>*Only common tests should be described solely by name; describe more complex techniques in the Methods section.*
☐	☒	A description of all covariates tested
☐	☒	A description of any assumptions or corrections, such as tests of normality and adjustment for multiple comparisons
☐	☒	A full description of the statistical parameters including central tendency (e.g. means) or other basic estimates (e.g. regression coefficient) AND variation (e.g. standard deviation) or associated estimates of uncertainty (e.g. confidence intervals)
☐	☒	For null hypothesis testing, the test statistic (e.g. F, t, r) with confidence intervals, effect sizes, degrees of freedom and P value noted<br>*Give P values as exact values whenever suitable.*
☒	☐	For Bayesian analysis, information on the choice of priors and Markov chain Monte Carlo settings
☐	☒	For hierarchical and complex designs, identification of the appropriate level for tests and full reporting of outcomes
☒	☐	Estimates of effect sizes (e.g. Cohen's d, Pearson's r), indicating how they were calculated

Our web collection on statistics for biologists contains articles on many of the points above.

Software and code

Policy information about availability of computer code

Data collection	No software was used to collect the data.
Data analysis	The open-source software R version 3.3.6 was used to perform the statistical analyses. The commercial software ArcGIS v. 2.2.3 was used to perform the GIS analyses.

For manuscripts utilizing custom algorithms or software that are central to the research but not yet described in published literature, software must be made available to editors/reviewers. We strongly encourage code deposition in a community repository (e.g. GitHub). See the Nature Research guidelines for submitting code & software for further information.

Data

Policy information about availability of data

All manuscripts must include a data availability statement. This statement should provide the following information, where applicable:

- Accession codes, unique identifiers, or web links for publicly available datasets
- A list of figures that have associated raw data
- A description of any restrictions on data availability

All data generated or analysed during this study are included in this published article (and its supplementary information files). The source data underlying the large-scale statistical analyses and plots shown in figures 1-3 and 5 are provided in Supplementary Data 1. The source data from the 2014 ecosystem survey, underlying the analysis and plot shown in Figure 4, is provided in Supplementary Data 2. Much of the fish survey data was extracted from the Swedish national database for coastal fish (for more information, see http://www.slu.se/kul).

Field-specific reporting

Please select the one below that is the best fit for your research. If you are not sure, read the appropriate sections before making your selection.

☐ Life sciences ☐ Behavioural & social sciences ☒ Ecological, evolutionary & environmental sciences

For a reference copy of the document with all sections, see nature.com/documents/nr-reporting-summary-flat.pdf

Ecological, evolutionary & environmental sciences study design

All studies must disclose on these points even when the disclosure is negative.

Study description	To assess temporal changes and spatial variability in recruitment of Eurasian perch (Perca fluviatilis), northern pike (Esox lucius) and three-spined stickleback (Gasterosteus aculeatus) along the western Baltic Sea archipelago coast, we first extracted field survey data on juvenile abundance from the Swedish national database for coastal fish (http://www.slu.se/kul). To cover the longest time period possible, and the full spatial range of two abiotic gradients know to affect juvenile fish composition (distance to offshore areas and wave exposure) we then searched for and included all additional datasets we could find (by searching online databases, contacting colleagues, and searching through physical archives). The final dataset included 13,077 samplings of juvenile fish conducted during 1979-2017, spread across 486 shallow coastal bays. Most bays (75%) were sampled once but 25% during 2-37 years, resulting in 842 bay-year combinations. Given that both absolute and relative abundances of the three species can shift locally between years (see manuscript and Fig. 2), we treated the 842 bay-years as independent replicates (N = 842).<br>To assess the relative importance of abiotic gradients and biotic interactions (focusing on predation and predator-prey reversal) for recruitment of perch, pike and three-spined stickleback we conducted an ecosystem field survey 32 shallow bays in 2014.
Research sample	In the large-scale field survey, each sample consisted of estimated density (number of individuals per unit surface area) of young-of-the-year (age 0+) Eurasian perch (Perca fluviatilis), northern pike (Esox lucius) and three-spined stickleback (Gasterosteus aculeatus). The sample estimates juvenile fish recruitment in each bay and year, respectively.<br>In the 2014 ecosystem field survey, we sampled both adult and juvenile fish in the 32 coastal bays. To ensure that the bays represented individual perch and pike populations, the bays were spaced >10 km apart or separated by natural fish movement barriers (land or deep, open water).
Sampling strategy	In the large-scale field survey, where we were restricted to using the data available, no statistical method was used to determine sample size; instead we included as many of the existing datasets on juvenile fish recruitment as possible. To reduce the influence of small-scale random variability, we only included surveys with at least 5 samplings per bay and year (the exact threshold being a trade-off between data availability and precision).<br>In the ecosystem field survey in 2014, 32 bays were sampled. The different variables (see below) were subsampled within each bay (n = 4-8, depending on variable), and averaged to form 1 replicate per bay. The sample size (N = 32) was deemed sufficient for statistical piecewise path analyses, if limiting the number of predictor variables to 7.
Data collection	Young-of-the-year juvenile coastal fish were sampled using low-impact pressure waves; a standard method in Baltic Sea juvenile fish monitoring. In short, an underwater detonation stunned or killed all small fish (2-20 cm) with a swim bladder within the blast radius (ca. 5m using the current detonation standard ; see manuscript Methods for details). The fish were then collected, identified and counted. The data used for the analyses of large-scale, long-term variability was collected by >15 fish monitoring programs and research projects conducted during the past 39 years. In most cases, there was no information available on who (individuals) performed the sampling.<br>In the ecosystem field survey in 2014, all juvenile fish sampling was conducted by J. Eklöf, J. Hansen, G Sundblad and Å. Austin (all certified to use explosives for scientific test fishing). The sampling of adult fish in May 2014 was conducted by J. Eklöf, J. Hansen, B.K. Eriksson, U. Bergström, S. Donadi, Å Austin. P. Jacobsson, G. Johansson, M. van Regteren, S. Skoglund, V. Thunell, andM. van Snoek. The sampling was conducted using Nordic survey gillnets, following the European Union standards for freshwater surveys (SS-EN 14757). The nets were 30×1.5 m and consisted of twelve 2.5 m panels with the following mesh sizes (in correct order): 43, 19.5, 6.25, 10, 55, 8, 12.5, 24, 15.5, 5, 35 and 29 mm knot-to-knot. In each bay, four to five nets were set at 1.5-3 m depth, >30 m apart and >10 meters from land and bay openings. The nets were set 16:00-19:00 and lifted 07:00-09:00 the day after. All caught fish were identified, counted and measured (total length, closest 1 mm).<br>The 2014 sampling of aquatic vegetation (in May and August) was conducted by a snorkeler (J. Hansen, G. Johansson, S. Donadi, Å Austin), noting the % bottom cover (0-100%) of individual species of aquatic macrophytes (plants, rhizoid macroalgae, drift macroalgae, etc.) in 6-8 stations per bay.<br>The 2014 sampling of zooplankton was conducted by J. Eklöf, J. Hansen, S. Donadi and Å Austin. A 25 cm diameter Epstein net with a 80 um mesh was pulled vertically from 0.7m above the seabed to the water surface. The zooplankton were collected in 4% formalin, and the procedure was repeated three times per 6-8 stations per bay.
Timing and spatial scale	The data included in the large-scale field survey was sampled during summer (in most cases, July-September) between 1979 and 2017. Spatially, the sampling covered a 1200 km stretch (birds distance) of the western Baltic Sea coastline. Most bays were sampled only once. Most of the surveys have been conducted after year 2000. We therefore included as many surveys as possible conducted during the period 1979-2000. The temporal and spatial extent of the data collection is described graphically in Extended data Fig. 9.
Data exclusions	In the dataset from the large-scale field surveys we excluded bay-years with <5 samplings to reduce the influence of random, small-scale variability in juvenile fish abundance. We also excluded 9 bays that occurred much further into the archipelago than the rest of the bays, and were only sampled after 2011 (because including them meant that temporal and spatial sampling extent were confounded).<br>In the dataset from the small-scale field survey in 2014, we initially included data from 32 bays. Initial data exploration using multiple regression showed that one bay was a clear statistical outlier due to 0 juvenile perch and pike present, generating too high statistical

	leverage (influence on linear relationships) for useful models, heteroscedasticity and non-normally distributed errors. Since juveniles had most likely already migrated out of the bay before sampling, the bay was excluded (resulting in N = 31). Removing this outlier greatly increased the fulfillment of test assumptions and the overall fit of the model (adjusted R2 increased from 0.17 to 0.37).
Reproducibility	Since no experiments were conducted, there were no attempts to repeat the study. However, the large-scale field survey consists of >20 individual ('replicated') surveys of juvenile fish, all demonstrating a clearly negative relationship between perch/pike and three-spined stickleback abundance and relative dominance.
Randomization	Since no experiment was conducted, no treatments could be allocated to any units. To cover the full gradients in the main predictor variables in the large-scale analysis (time, distance to open sea, wave exposure) we included as many surveys as possible, and especially sought to include those that had sampled the low and high ranges of the gradients (e.g. bays close to offshore areas or far into the deepest archipelago). To assess the potential influence of covariates on relative predator dominance, we assessed the potential influence of four factors known to also influence juvenile fish (water temperature, salinity, depth and turbidity; see Methods for details).
Blinding	Since all data was collected using field surveys, no blinding was possible.

Did the study involve field work? ☒ Yes ☐ No

Field work, collection and transport

Field conditions	In the large-scale, long-term field survey, data on water temperature and salinity was included when sampled by each respective monitoring program/research project.<br>In the small-scale field survey in 2014, data on water temperature and salinity was measured in each bay during both sampling periods (spring and late summer).
Location	The data used in the large-scale analyses were sampled in 486 shallow coastal bays situated along a 1200 km stretch of the Swedish Baltic Sea coastline (55-65°N), from Blekinge in the south to Kalix in the north. Average water depth was 1.7m.<br>The small-scale field survey in 2014 was conducted in 32 bays situated along a 360 km stretch of the central Swedish Baltic Sea coastline (57.8-60.3°N) (see extended data fig. 7).
Access and import/export	All sampling for the large-scale, long-term survey was conducted in accordance with (at the time of sampling) existing rules and regulations. For the data extracted from Swedish national coastal fish database (KUL) we have no information on approving authorities.<br>For the sampling in the 2014 field survey (which the authors conducted), we first received oral approval (by phone call) from each respective water property owner (private individuals, Länstyrelsen Stockholm, Länstyrelsen Sörmland, Upplandsstiftelsen) to conduct the sampling in the 32 bays. To transport and use explosives for the low-impact pressure wave sampling, J. Eklöf received status of manager of explosive work by the Vice Chancellor of Stockholm University (SU FV-2.7-1830-14). The transport and use of explosives was approved by Södertörns brandskyddsförening (Dnr 2014-24-17), Storstockholms brandförsvar (Dnr 317-862/14), Brandkåren Attunda (Dnr 304.2014.01113) and the Police authority in Stockholm county (Dnr: 523A263.495/2014). All permits were issued in March-April 2014.
Disturbance	The method used to sample juvenile fish (underwater detonations) stuns and/or kills all small fish (2-20cm) with a swim bladder. To reduce the amount of disturbance on wildlife in the 2014 survey, a maximum of 8 samplings was done per bay (to make sure that <1% of the bay surface area was affected).

Reporting for specific materials, systems and methods

We require information from authors about some types of materials, experimental systems and methods used in many studies. Here, indicate whether each material, system or method listed is relevant to your study. If you are not sure if a list item applies to your research, read the appropriate section before selecting a response.

Materials & experimental systems

n/a	Involved in the study	
☒	☐	Antibodies
☒	☐	Eukaryotic cell lines
☒	☐	Palaeontology
☐	☒	Animals and other organisms
☒	☐	Human research participants
☒	☐	Clinical data

Methods

n/a	Involved in the study	
☒	☐	ChIP-seq
☒	☐	Flow cytometry
☒	☐	MRI-based neuroimaging

Animals and other organisms

Policy information about studies involving animals; ARRIVE guidelines recommended for reporting animal research

Laboratory animals	The study did not involve laboratory animals
Wild animals	The study focused on young-of-the-year (age 0+) fish from three species: Eurasian perch (Perca fluviatilis), northern pike (Esox

Wild animals	lucius) and three-spined stickleback (Gasterosteus aculeatus). The fish were sampled using low-impact pressure waves induced by underwater detonations; a standard method in Baltic Sea monitoring of coastal fish recruitment. In short, an underwater detonation generated a pressure wave which stuns or kills all small fish with a swim bladder (2-20cm length). The fish were collected (by swing nets from boat and/or by snorkelers) and then identified and counted on the boat. The individual fish were not sex-determined. Injured individuals were directly euthanized using (at the time) recommended and approved methods (MS-222, destroying the brain, breaking the neck). Since we did not conduct most of the historical sampling included in the data extracted from the national database, we cannot describe exactly how the fish were euthanized in each respective sampling.<br><br>In the 2014 small-scale survey, we sampled juvenile (young-of-the-year) fish using low-impact pressure waves, and adult fish using standardized gill (see above and manuscript for details). Individual juveniles that were only stunned were directly transferred to buckets with seawater, and (if showing natural behavior within 30 min of capture) released. Alive but injured individual fish were directly euthanized using tricaine methanesulfonate (MS-222).
Field-collected samples	The sampled fish were released live or (if injured) euthanized in the field, using approved methods (see above).
Ethics oversight	The numerous fish samplings providing data for the long-term, large-scale analyses were conducted by >15 different monitoring programs and research projects during a 39-year period. No data is available in the KUL database on which exact organizations that approved or provided guidance on each study protocol.<br>The fish sampling for the smaller ecosystem field survey we conducted in year 2014 was evaluated and approved by the ethical board on animal experiments of the County court of Uppsala, Sweden, permit C 139/13.

Note that full information on the approval of the study protocol must also be provided in the manuscript.
